# Collagen Derived from Fish Industry Waste: Progresses and Challenges

**DOI:** 10.3390/polym15030544

**Published:** 2023-01-20

**Authors:** Zahra Rajabimashhadi, Nunzia Gallo, Luca Salvatore, Francesca Lionetto

**Affiliations:** 1 Department of Engineering for Innovation, University of Salento, Ecotekne Center, 73100 Lecce, Italy; 2 Typeone Srl, Muro Leccese (LE), 73100 Lecce, Italy

**Keywords:** fish collagen, fish industry waste, collagen extraction, nano collagen, sustainability

## Abstract

Fish collagen garnered significant academic and commercial focus in the last decades featuring prospective applications in a variety of health-related industries, including food, medicine, pharmaceutics, and cosmetics. Due to its distinct advantages over mammalian-based collagen, including the reduced zoonosis transmission risk, the absence of cultural-religious limitations, the cost-effectiveness of manufacturing process, and its superior bioavailability, the use of collagen derived from fish wastes (i.e., skin, scales) quickly expanded. Moreover, by-products are low cost and the need to minimize fish industry waste’s environmental impact paved the way for the use of discards in the development of collagen-based products with remarkable added value. This review summarizes the recent advances in the valorization of fish industry wastes for the extraction of collagen used in several applications. Issues related to processing and characterization of collagen were presented. Moreover, an overview of the most relevant applications in food industry, nutraceutical, cosmetics, tissue engineering, and food packaging of the last three years was introduced. Lastly, the fish-collagen market and the open technological challenges to a reliable recovery and exploitation of this biopolymer were discussed.

## 1. Introduction

In order to exploit natural resources as much as possible, a long-term plan titled “Blue Growth” was approved by the European Commission and has been implemented to pay particular attention to fish resources in order to preserve the environment from industrial pollution. The enormous amount of valuable protein that could be extracted [1,2,3,4,5] (about 30–40% of the total weight), is one of the most appealing aspects of seafood by-products. More than 20 million tons of them are produced annually from the fish tissues that are discarded as waste, including fins, heads, skin, and viscera [6,7,8]. Because of their elevated protein content, absence of disease transmission risks, high bioactivity, and less considerable religious and ethical restrictions, the use of fish by-products as a new source of collagen has drawn increasing attention [9,10,11].

The importance of both aquaculture and fishing to food security is expanding continuously, particularly in light of the rising global fish production and the United Nations’ 2030 program of sustainable development [12]. Approximately 70% of fish and other seafood are processed before being sold, resulting in enormous amounts of solid waste from processes such as beheading, de-shelling, degutting, separating fin and scales, and filleting [13,14]. More than half of the weight of fresh fish becomes by-products of the fish industry. Most of these by-products are buried or burned, causing environmental, health, and economic issues. A minor portion are employed as inexpensive ingredients in animal feeds. Fish waste is a rising problem that requires quick, creative methods and solutions. Numerous initiatives and programs have been performed globally to prevent food waste. In addition to reducing the cost of waste disposal, investing in waste from the fish industry can offer the opportunity to recover other important substances such as oils, proteins, pigments, bioactive peptides, amino acids, collagen, chitin, gelatin, etc. [15,16,17].

More than two decades ago, research on the extraction of collagen from fish waste started to be conducted. Collagens are one of the most abundant proteins in animals, which are found in the extracellular matrix of connective tissues, including skin, bones, tendons, ligaments, cartilage, intervertebral discs, and blood vessels [18]. Collagens are not only implicated in tissue architecture maintenance and strength, but they also cover regulatory roles (i.e., through mechano-chemical transduction mechanisms) during tissue growth and repair [19,20]. Thanks to their nature, collagens are intrinsically bioactive, biocompatible, and biodegradable [21]. Hence, collagens are valued as the most commonly required and used biomaterials in many fields, including medical, cosmetic, nutraceutical, food and pharmaceutical industries in the forms of injectable solutions, thin substrates, porous sponges, nanofibrous matrices, and micro- and nano-spheres [22,23,24,25]. Recent studies revealed many similarities in the molecular structure and biochemical properties between collagen derived from fish and mammalian sources, despite the fact that fish collagen typically has a lower molecular weight and lower denaturation temperature than mammalian collagen [8,12,20,22,24,26,27,28]. Various extraction techniques for fish collagen have been developed depending on the selected tissue type and fish species. Hence, a considerable collection of literature has been developed on this subject [29,30,31]. Only in the past five years have researchers concentrated on innovative materials with improved characteristics in addition to developing extraction techniques for mass manufacture.

**Figure 1:**
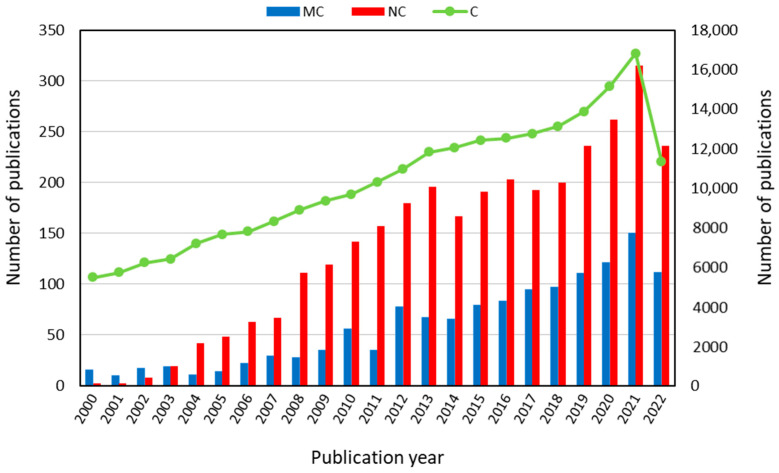
Increasing research interest in fish collagen (MC) and nano collagen (NC) compared with collagen (C), according to scientific papers analyzed by publication year in the last twenty years up to 2022 (from Scopus database: www.scopus.com, accessed on 15 September 2022).

Collagen nanotechnology has a bright outlook because science in this area is always progressing and will continue to do so in the future. Nano collagen is ordinary collagen that has been sized down to a nanometer scale [32,33]. According to its nano-scale-based technology, which offers a high surface-area-to-volume ratio, an optimal penetration into wound sites and higher cell interaction is enabled. [34]. Moreover, nano collagen has the ability to deliver drugs and to supply a durable microenvironment at wounded sites to promote cellular regrowth and healing [35]. Collagen nanotechnology still presents many shortcomings, including the fact that only a small minority of therapeutic compounds have received commercial approval and that there are still numerous unsolved problems [32]. The complexity of pathophysiological symptoms and the lack of data on its real physiological effects is a further challenge for nanotechnology. Despite these downsides, nanotechnology is still a growing trend, with a huge amount of unrealized potential. This gives rise to the expectation that further research will assist in minimizing these downsides, leading to the creation of secure and efficient nano-based systems. In order to create approved therapeutic agents that take advantage of nanotechnology, additional research and studies must be performed [32]. Indeed, Figure 1 reveals the continuous increasing research interest on collagen, fish collagen, and nano collagen investigation in the last twenty years. In particular, it appears clear that there has been a significant increase in scientific works in the last five years. Nano collagen can be used for a variety of improvements and treatments, such as bone grafting, drug delivery, nerve tissue formation, vascular grafting, articular cartilage regeneration, cosmetics, and wound healing [21,22,36]. It is clear that nano collagen is a progressed type of nanotechnology; thus, further investigation must be attempted to advance this technology with the expectation that, in the future, nano collagen scaffolds will be more widely available [37].

This review aims to provide an overview of recent investigations into fish collagen, with a particular focus on its characteristics, types, and extraction methods, and, finally, on the valuation of fish industry waste for the preparation of biopolymers for various applications areas. Among others, fish-collagen application in the medical, pharmaceuticals, food, and cosmetic sectors are discussed.

## 2. Collagen: Structure and Properties

Collagens represent about 30% of a mammal’s weight [18,38]. Based on the historical order of their discovery, 28 types of collagens—type I through type XXVIII—have been identified and described up to the current day [39]. The oldest collagen identified to date was found in the soft tissue of a fossilized *Tyrannosaurus rex* bone that dates back 68 million years [40,41].

**Figure 2:**
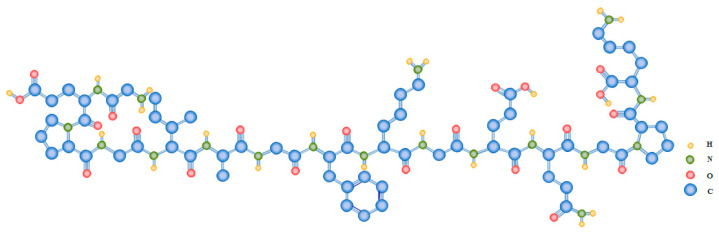
Exemplary amino acid repetition of the triplet (Gly-X-Y)n characteristic of type I collagen.

The molecular organization of collagens is highly variable, notwithstanding their general triple-helical structure and the triplet (Gly-X-Y)n repetition, where X and Y can be any amino acid, although proline and hydroxyproline are the most frequent occupants of these locations (Figure 2) [42,43]. Collagen’s unit is composed of three α-chains, the amino acid composition of which varies among collagen types. Furthermore, function and distribution in tissues play a role in the diversity of collagen, as well as molecular and supramolecular organization, such as occurrence and length of triple helical domains, packing of the triple helices, etc. [27,44].

The most prevalent and thoroughly studied types of collagens are type I (almost present in all tissues and organs), type II (present in the cartilage, vitreous body, and nucleus pulposus), and type III (present in the skin, blood vessels, lungs, liver, and spleen) [45], which are used in tissue engineering and reconstructive medicine as well as in the pharmaceutical industry as compounds that extend the effects of drugs. Types I, II, and III collagens, especially type I, are also used as plastics in medicine and cosmetology. Type I collagen represents over 70% of the entire collagen family and makes up more than 90% of the collagen in the human body. It is mainly found in connective tissues such as body joints, cartilages, bones, sclerae, ligaments, tendons, intervertebral discs, corneas, adventitia of blood vessels, skin, and most hollow organs including gastrointestinal and genitourinary tracts [24,39,46]. In contrast, types II, III, and IV collagen are frequently seen. Type II collagen, for instance, is a structurally important part of the hyaline cartilage that lines the adult’s articular surfaces in addition to being present in other tissues including the intervertebral disc’s nucleus pulposus and the retina, sclera, and lens of the eye. Skin, lungs, intestinal walls, and blood vessel walls all contain type III collagen.

Type I collagen is composed of three polypeptide chains, two identical α1(I) chains and one α1(I) chain, each of which has roughly 1000 amino acid residues [47]. Hydroxylation of proline residues is a typical post-traditional modification of type I collagen that accounts for about 11–14% of amino acid residues and it is commonly used as a marker to detect and quantify collagen in tissues [48,49]. Whereas proline and hydroxyproline are essential for maintaining the triple helical structure under physiological conditions by forming hydrogen bonds that inhibit uncontrolled rotation, glycine is critical for packing the three helices [50,51].

**Figure 3:**
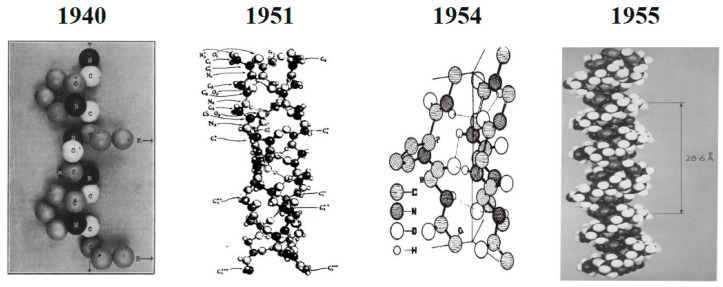
Changes in the proposed structure of type I collagen from the beginning and its modification to the final accepted structure. Adapted from [52]. Reproduced from [51] with permission from springer Nature, 1940. Reproduced from [53] with permission from springer Nature, 1954. Reproduced from [54] with permission from Elsevier, 1955.

The idea that the type I collagen molecule is made up of a single extended polypeptide chain with all amide bonds was brought forward by Astbury and Bell in 1940 [51]. In 1951, Pauling and Corey provided the correct structures for the α-helix and β-sheet [52]. In that proposal structure, three polypeptide chains were connected in a helical configuration by hydrogen bonds. These hydrogen bonds necessitated the production of two of the three peptide bonds and involved four of the six main chain heteroatoms inside each amino acid triplet [52]. The collagen triple helix structure was reconstructed in 1954 by Ramachandran and Kartha as a right-handed triple helix of three staggered, left-handed helices with one peptide bond and two hydrogen bonds within each triplet [53]. In 1955, this structure was improved by Rich and Crick, North, and Colleagues thanks to which the triple-helical structure that is still used today was unveiled. This structure has helical symmetry and just one crosslinking hydrogen bond per triplet [54]. Changes in the proposed structure of collagen from the beginning and its modification to the final structure accepted by the scientific community are shown in Figure 3.

**Figure 4:**
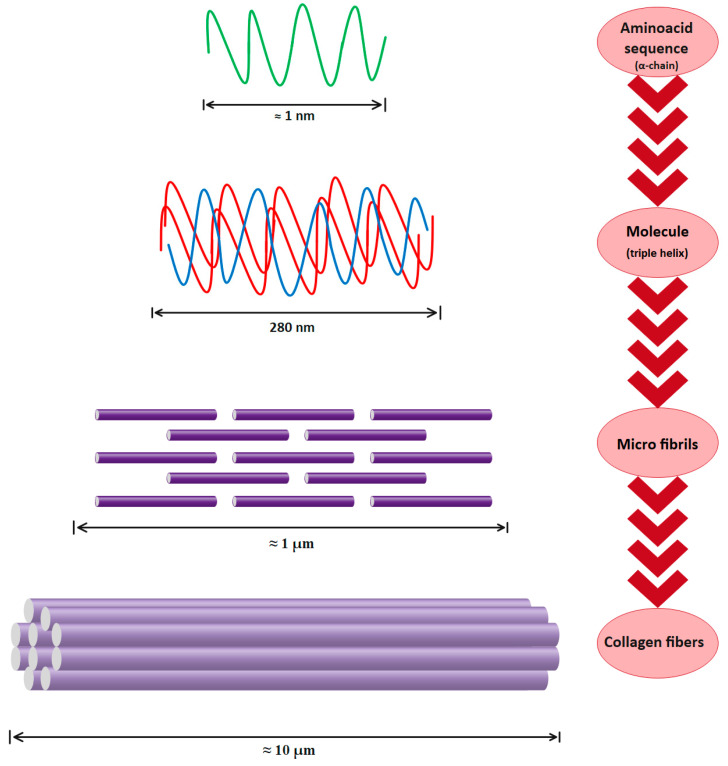
Type I collagen hierarchical organization.

As is shown in Figure 4, three polypeptide α-chains form the trimeric molecule that represents the type I collagen unit (length ≈ 300 nm, diameter ≈ 1.5 nm). Three parallel, left-handed polyproline-II helices are arranged in a right-handed bundle [55,56]. Multiple collagen units are assembled into fibrils (length ≈ μm, diameter ≈ 100 nm) and then fibers (length ≈ mmm diameter ≈ 10 μm) with dimensions and orientation that are strictly tissue-dependent [28,57].

Thus, type I collagen is a hierarchically organized protein. The primary structure of type I collagen consists of three α helices: two identical α1(I) and one α2(I) helices of approximately 1000 amino acids and a molecular weight of about 130–140 kDa and 110–120 kDa, respectively. The collagen molecule has a triple helical part and two non-helical parts at both ends (called telopeptides), with a molecular weight of 300–400 kDa, a length of 280 nm, and a width of 1.4 nm [58,59]. The secondary structure consists of each of these chains twisted in the form of a left-handed helix with three amino-acid repetitions in each turn. The tertiary structure, the inflexible structure, is created when the chains are then twisted three times around one another. Finally, in the quaternary structure, collagen molecules assemble into fibrils and then fibers. Because of the intermolecular and intramolecular interactions, this collagen organization is very stable [25,60]. Obviously, the collagen structure’s stability is directly dependent on its chemical composition. For instance, the triple helix of collagen grows stronger as the percentage of amino acids is higher, such as proline and hydroxyproline. The pyrrolidine rings are directly responsible for the polypeptide chain’s movement reduction [22,61]. Preservation of collagen’s structural integrity results in an improvement in physical properties, an increase in thermal stability, and a decrease in the denaturation temperature [62,63,64].

Theoretical examination of the mechanical characteristics of collagen at several levels, including the main monomer, individual collagen fibrils, and collagen fibers, is possible by studying collagen’s structured nature. Studying main monomers and fibrils made from type I collagen has likely provided the most direct measurements of the mechanical properties of collagen. Over the recent decades, researchers have used a variety of biophysical and theoretical methods, and recent developments in the Atomic Force Microscopy (AFM) approach have made it possible to perform more accurate evaluations [65]. According to estimates, the fracture strength of individual collagen triple helices is 11 GPa, which is much higher than that of collagen fibrils, which is 0.5 GPa [66]. This difference makes sense because, whereas the breaking of a fibril does not always entail the breakdown of covalent bonds, the breaking of individual collagen triple helices necessitates the unwinding of the triple helix and ultimately breaking of the covalent bonds [67]. In contrast to dehydrated type I collagen fibrils from mammalian sources, which have a Young’s modulus of about 5 GPa according to AFM tests, individual collagen triple helices monomers have a Young’s modulus between 6 and 7 GPa. Because collagen fibrils are anisotropic, another crucial measure of a collagen fibril’s strength is its shear modulus, which determines stiffness [68].

Furthermore, AFM indicated that the shear modulus of dehydrated fibrils of type I collagen from mammalian sources is between 30 and 35 MPa. These fibrils’ shear modulus was drastically decreased by hydration, but was increased by cross-linking. It is important to note that while some cross-linking is beneficial for the mechanical qualities of collagen fibrils, excessive cross-linking causes collagen fibrils to become highly brittle, which is a common sign of aging [69]. Investigation of the mechanical properties of collagen fibrils demonstrated that the length of the individual collagen triple helices monomer has been chosen by nature in a way to maximize the strength of the produced collagen fibril through effective energy dissipation. Simulations indicate that individual collagen triple helices monomers either longer or shorter than the length of a type I collagen triple helix, which is 300 nm, would form collagen fibrils with low mechanical properties [62]. The thermal and structural stability of the collagen triple helix is strongly influenced by the chemical composition of amino acid and its type, which is caused by the type of animal and the living conditions. Indeed, hydroxyproline stabilizes and strengthens the collagen structure [70]. In addition to preserving collagen’s structure and enhancing its mechanical properties, hydroxyproline also plays an important role in its thermal stability. The denaturation temperature and denaturation enthalpy of collagen increases due to the presence of the hydroxyl group in hydroxyproline and the bonding with the pyrrolidine ring. The quantity of hydrogen bonds formed between hydroxyproline and pyrrolidine significantly influences the increment in enthalpy of denaturation. Therefore, the triple helix does have greater thermal stability the more water molecules that there are surrounding it [25,71].

One of the most basic roles of collagen in the body is to provide connective tissues with stability, structure, and resistance to stresses [19,20]. Moreover, collagen has the ability to manage a wide range of nonstructural activities, including cell proliferation, migration, differentiation, and communication [60,72].

## 3. Fish Collagen

Collagen sources, types, pre-extraction conditions, and process methods are the main parameters that determine extracted product properties, including molecular weight of the peptide chain, amino acid composition, molecular structure, solubility, and functional activity. Although native type I collagen could be extracted from different mammalian sources, the main source of extraction is bovine due to availability and biocompatibility. [73]. There are other alternative sources for extracting type I collagen, among which pig, horse, sheep, and rat can be mentioned [74,75,76,77]. It is possible to obtain mammalian collagen from a wide range of tissues, notably skin, bones, tendons, lung tissue, and connective tissues. Due to some restrictions in terms of health, cultural, social, and religious issues that are implied by traditional sources, research has concentrated on the development of a new source of extraction. Various resources from the sea, including vertebrates as well as invertebrates, have been studied and considered as collagen extraction sources. In particular, several fish species (e.g., *Rachycentron canadum, Esoxlucius, Spotless smooth hound, Sciaenops ocellatus, Sardinella fimbriata, Coryphaena hippurus, Alaska pollock, Takifugu flavidus, Pacu, Labeo rohita, Labeo catla, Tuna, Thunnus obesus, Scomber japonicus, Gadus morhua, Prionace glauca, Cichla ocellaris, Cyprinus carpio, Oreochromis niloticus, etc.*) aquatic reptiles (such as the soft-shelled turtles), sponges, corals, octopuses, squids, starfish, jellyfish, cuttlefish and sea cucumbers, sea anemones, sea urchins, mussels, and shells were considered.

**Figure 5:**
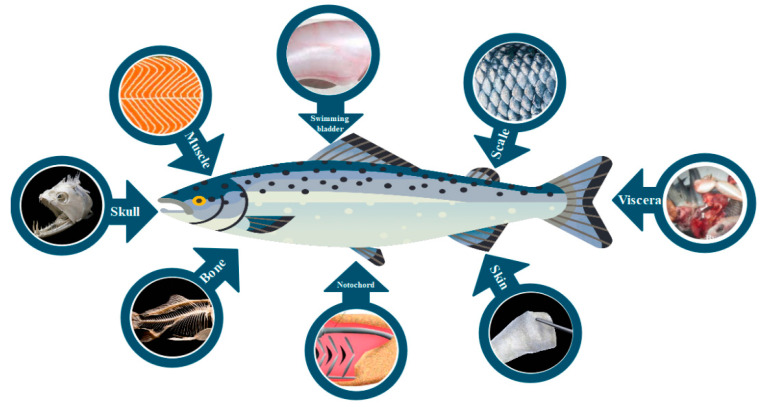
By-products of fish as potential sources of collagen extraction.

Skin, scales, bones, skull, swimming bladder, and remaining viscera, are by-products of fish that may be used as sources of collagen (Figure 5). Among all fish by-products, skin traditionally has been reported as the best source of fish collagen extraction [12,78,79,80,81].

**Figure 6:**
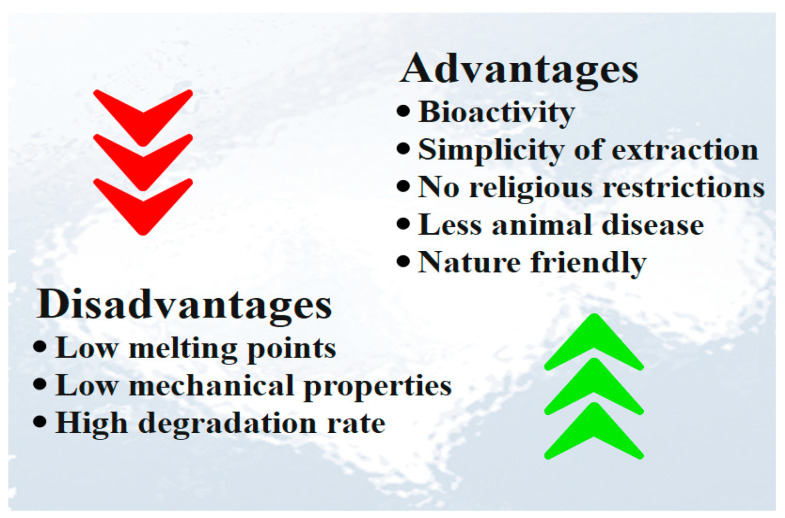
Advantages and disadvantages of fish collagen.

Fish collagen physicochemical properties were found to be similar to mammalian collagen, but with some advantages such as (1) capability of purification and extraction; (2) aquaculture and accessibility to fishing by-product; (3) lower risk of disease transmission compared to mammalian collagen due to high ontogenetic difference between fish and humans; (4) lack of religious and cultural limitation; (5) slightly different chemical composition; (6) low viscosity; (7) non toxicity; (8) reasonable homeostatic properties; (9) bio-resorbability; (10) more simple extraction method; (11) more adaptable and metabolic compatibility; and (12) minimal inflammatory response (Figure 6) [82,83,84,85]. Although fish collagen has several advantages, it suffers from several disadvantages such as low denaturation temperature, low mechanical properties, and high degradation rate [78]. The major drawback of fish collagen compared to mammalian collagen is the lower denaturation temperature, which limits its medical applications [70]. During denaturation, collagen turns into gelatin, where the hydrogen bonds that support the helical structure are partially or completely destroyed, and it loses its structural role and its conformation-related biological activity [42,43]. The second main drawback of fish-derived collagen is its low mechanical resistance which limits its applications. Many efforts have been made to improve its mechanical properties and degradation profiles, including chemical or enzymatic cross-linking [86,87]. The different advantages and disadvantages of fish collagen are shown in Figure 6.

## 4. Collagen Extraction Methods

Due to the enormous diversity of collagen types, it is challenging to design a standard extraction procedure for collagen from various tissues. However, the collagen extraction process usually consists of around five main steps (Table 1): (i) tissue separation and purification; (ii) tissue size reduction; (iii) non-collagenous components elimination; (vi) collagen extraction through acid and/or enzymatic treatment; (v) and, finally, recovery using salt precipitation. The extraction procedures start with the removal of unneeded portions. Fish by-products are then reduced in size to facilitate the following step which is the removal of non-collagen proteins, lipids, pigments, cell remnants, and minerals. Afterwards, collagen is extracted using an acidic treatment, followed by an optional enzymatic treatment, before being recovered using salt precipitation, dialyzation, and lyophilization. All these steps are performed at about 4° C to 10 °C, to prevent collagen denaturation [88,89,90].

The conventional process for collagen extraction, based on acid and/or enzymatic methods, has been improved in recent research. The fish ecosystem, the belonging tissue, and the method of extracting collagen from a fish source have a direct effect on the number of remaining impurities [83,91]. In the following, various sources, methods, advantages, and disadvantages of each method and effective parameters in the extraction of fish collagen have been investigated.

Fish tissues require special treatment before being recovered from fisheries and aquaculture byproducts, including washing with water and sodium chloride to remove impurities and lipids and milling the skin to increase its contact surface with the liquid phase [92]. Following the removal of contaminants and non-collagenous proteins using sodium hydroxide, hydrogen peroxide, calcium hydroxide, or a combination of these, the material is submerged in alkaline solutions, with butyl alcohol (10%) used to remove oily components [93,94].

The real extraction step is based on the solubility of the collagenous molecule taken after the pretreatment. The most common treatments are saline, acid, and/or enzymatic. The saline treatment employs neutral salt, such as sodium chloride and/or guanidine hydrochloride, for precipitation-based extraction, which, among its drawbacks, has a low extraction yield [95]. Once the sodium chloride concentration has been gradually raised through adding NaCl, the collagen is separated. It was shown that the basic salt extraction is ineffective after testing a number of collagen isolation techniques. Additionally, raising the salt content will enhance the ionic power of the resulting solution and boost the solubilization capacity. The ultimate yield is extremely low since, in normal tissues, the amount of neutral salt-soluble collagen is typically insignificant [96,97].

In the acid treatment, several acid types could be employed, such as acetic acid, lactic acid, citric acid, hydrochloric acid, formic acid, sulfuric acid, and tartaric acid [83]. Obviously, this method can solubilize collagen more effectively than basic salt extraction, but it is still only effective on young and uncross-linked collagen [97]. Collagen type I derived from fish skin is often extracted using an acidic treatment with acetic acid, hydrochloric acid, or phosphoric acid. However, this extraction method can be performed using either acids or alkali. These extraction techniques are extremely corrosive and, after neutralization, result in a high salt content. The pH value will influence the electrostatic interaction and structure depending on the acid’s concentration. It establishes the ability of animal tissue to be extracted and dissolved [28,98,99].

Enzymatic treatment involves the use of enzymes, such as collagenase, papain, or pepsin [100,101]. Enzymatic hydrolysis has emerged as the best method for collagen extraction from fish because it tends to eliminate the non-helical extremities and increase the solubility of collagen molecules and, thus, increase the extracted material yield [102,103]. The potential for irreversible denaturation of the collagen structure by enzymatic digestion during this procedure could either be a drawback or not, since it could be used for the production of several collagen formulations with different hierarchical organization levels that will have different bioactivity profiles. A more effective collagen extraction method was obtained by integrating both the acidic and enzymatic treatments. The collagen molecule is affected by enzymes, which make it more soluble in an acidic media [61,104].

**Figure 7:**
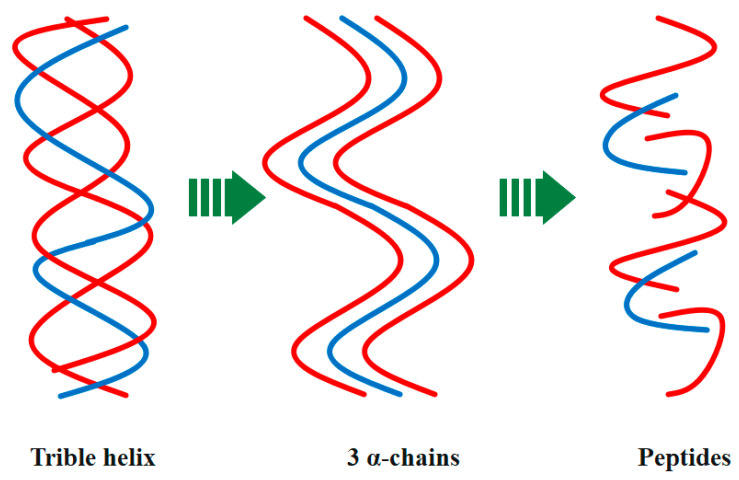
Representative scheme of type I collagen denaturation into low-molecular-weight peptides (red and blue).

Figure 7 shows that the denaturation of native collagen results in the formation of randomly coiled α-chains. Thermal treatments above collagen denaturation temperature can be used to obtain them. Proteolytic enzymes are able to hydrolyze the polypeptide chains in shorter polypeptide sequences. The final outcome is typically referred to as hydrolyzed collagen that is made up of short, low-molecular-weight peptides. The kind and level of hydrolysis, as well as the different type of enzyme used in the process, all affect collagen properties and functional activity [105,106,107,108].

The molecule is not altered when collagen is extracted using ultrasonic as a substitute method; instead, this helps the enzymatic process [109,110]. This method can be used to produce higher collagen yields in shorter extraction durations. This approach is more effective than the traditional one because it increases mass transfer by opening the collagen fibrils, permitting acid and/or enzymatic hydrolysis, and subsequently improving the extraction yield [109,110,111].

**Figure 8:**
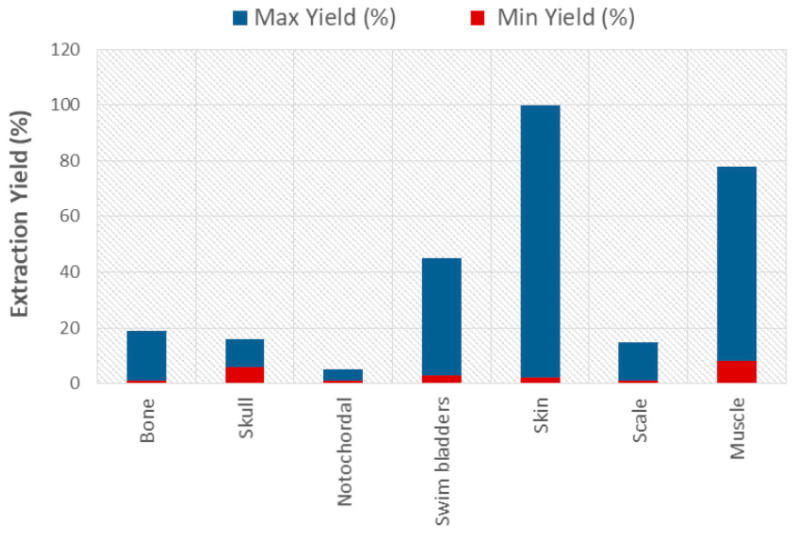
Yield of collagen obtained from fish sources [8].

Electro dialysis, a quick, effective, and affordable approach, was employed instead of traditional dialysis to boost extraction efficiency and process speed [112]. Isoelectric precipitation is a method frequently used to separate protein biomolecules, which can be used in collagen extraction from fish sources [113,114]. Thermal processing, or treating the protein to high pressure and temperature, constitute further extraction techniques. There is a subcritical water level (SCW) used in thermal processing, which can be found at pressures lower than 22 MPa and temperatures between 100 °C and 374 °C [97,115]. Figure 8 shows that the yield of collagen obtained varies from 0.05% to 94.4% [8].

Table 2 lists some recent studies on various techniques for collagen extraction from different fish sources. The primary objectives of introducing new methods during the collagen extraction phases are to shorten the extraction process time, energy, and chemicals compared to traditional methods. The efficiency of collagen extraction methods from animal by-products depends on the extraction source, age, and type of animal, as well as the condition of the processed by-products and the technology employed. 

## 5. Collagen Applications

**Figure 9:**
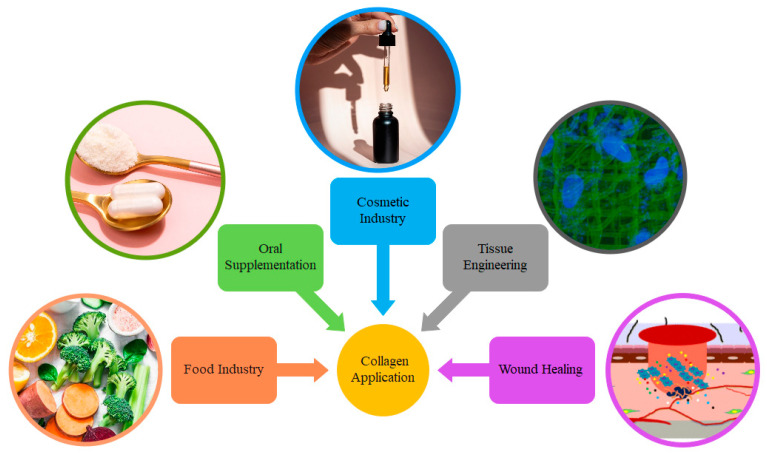
Application of fish collagen in different industrial fields.

Given its outstanding biocompatibility and biodegradability, low cytotoxicity, elevated versatility, significant therapeutic loading, affordability, lack of need for a multistep extraction procedure, high digestibility, and ease of absorption and distribution in the human body, fish collagen is even more frequently used in a many industrial areas [129,130]. Besides aforementioned advantages, it has a decreased viscosity in aqueous solution, low allergenicity, transparency, good solubility and dispersibility (i.e., uniform distribution in solution), emulsifying ability, and processability in different kinds of products such as powder, foam, and film [131,132]. Thus, throughout many different industrial sectors, including biomedical, pharmaceutical, food, cosmetic, and leather industries, type I collagen is widely employed, as presented in Figure 9. Some of these applications are mentioned below. For niche but promising applications in energy storage devices, the authors referred to a recent review [133].

### 5.1. Food Industry

In the past, collagen has been used to prepare a variety of goods, including meat products, drinks, soups, and others [123,129]. It aids in enhancing and maintaining their physical, chemical, and sensory qualities. Compared to patties made without fish collagen, those prepared with fish collagen have a higher protein percentage, reduced fat content, comparable sensory acceptance, and better texture. Even in processed foodstuffs including sausages, sausage rolls, ham, hotdogs, and hamburgers, collagen has replaced half-content pork fat leading to enhanced hardness and chewiness, better stability after cooking, and a higher water-holding capacity. Additionally, fish collagen can be added to drinks such as natural fruit juice, to enhance their nutritional and functional qualities due to their greater protein content, bioavailability, moderate viscosity, and excellent water solubility [134,135,136,137,138]. More recently, studies are ongoing on the use of fish (minced fillet) waste in the manufacturing of foodstuffs [139].

### 5.2. Nutraceuticals

Collagen plays a crucial role in tissue and organ development, maintenance, and healing. The loss of collagen in the body begins at the end of the second decade of life and reaches 1% per year by the end of the fourth decade. This process continues until the eighth decade, when the body has lost about three quarters of its collagen compared to the youth. Additionally, other factors such as diseases, improper diet, alcoholism, and smoking accelerate this process [140,141,142].

The largest apparatus in the human body is the integumental system, which is primarily made of proteoglycans, hyaluronic acid and elastic fibers, and collagens (mainly types I, III, V; types IV, VI, VII to a minor extent). Natural aging involves changes in the human body: the skin deteriorates morphologically, structurally, and functionally; collagen levels decline; and elastin fibers encourage the development of wrinkles. In the dermis, collagen has a double role: i) to serve as a building block for the formation of newly synthetized collagen and elastin fibers; ii) to interact with receptors on the fibroblasts’ membrane to promote the synthesis of new collagen, elastin, and hyaluronic acid [143]. Considering that collagen peptides have antioxidant and antibacterial properties and vary in quality depending on the technique of extraction, they can be employed as a component in functional dietary supplements. In view of the fact that collagen oral supplementation reaches the deeper layers of the skin and improves skin physiology and appearance by enhancing hydration, elasticity, firmness, wrinkle reduction, and skin regeneration, oral collagen supplementation has gained popularity in recent years [123,144]. Many studies have concluded that hydrolyzed fish collagen applied as food supplement is able to provide positive effects on skin appearance with enhanced water-holding capacity, moisture absorption, retention, anti-aging, and anti-melanogenic effects [59,145].

Skin condition changes brought on by aging are a crucial concern for preserving the quality of life. As a result, the public is interested in dietary supplementation’s ability to treat skin disorders. Naoki Ito postulated that, by elevating the plasma growth hormone, a supplement blend comprising ornithine and fish-derived collagen peptide could enhance skin conditions [146]. In this regard, two groups of volunteers used a supplement or identical placebo for two months. Skin condition, including elasticity and transepidermal water loss, as well as growth hormone levels, was significantly improved in the first group. The combination of amino acids in collagen hydrolysate, known as a safe nutraceutical, stimulated the production of collagen in the extracellular matrix of cartilage and other tissues. Porfírio performed research on the action of collagen hydrolysate in bone and cartilaginous tissue and its therapeutic use against osteoporosis and osteoarthritis, discovering a connection between the maintenance of bone strength and composition, as well as cartilage cell development and proliferation, and the administration of various doses of collagen hydrolysate [147]. This study concluded that hydrolyzed collagen has a protective effect on articular cartilage, and especially helps with symptomatic pain reduction considering the ability to raise bone mineral density [147]. Therefore, it has a good therapeutic effect on osteoporosis and osteoarthritis.

### 5.3. Cosmetics

As mentioned in the previous section, the role of collagen in the body is very important because it helps the skin, the largest organ of the human body. The skin protects the organism from external damage, regulates temperature, and performs other body functions. Over the years and in the process of aging, the amount of collagen in the skin decreases and this causes its morphological, structural, and functional deterioration. In fact, the presence of elastin fibers causes lines and wrinkles and shows aging. Controlling skin aging is a challenge in the cosmetic industry, but the use of collagen has been proven to be an alternative solution to reduce the effects of aging. In the studies that have been conducted, fish collagen has shown the capacity to retain water, absorb moisture, and retain it again, which can have anti-aging effects on the skin and can be used as a potential active ingredient in skin-care products [148,149,150,151].

### 5.4. Tissue Engineering and Regenerative Medicine

Historically, tissue engineering is based on the combination of scaffolds, cells, and signals. The term ‘scaffold’ is usually referred to as a temporal substitute that should structurally support tissue formation and provide the appropriate environment for cell migration, proliferation, and differentiation, and hence for repairing processes. The prevalence of collagen in human tissues and the important role it plays in the extracellular matrix make it a natural choice for its employment as raw material in the development of implantable devices for tissue engineering and regenerative medicine applications. Common application areas include bone, vascular tissue, skin, cartilage, corneal tissue, oral mucosa, and dental regeneration [26,73,152].

Numerous studies demonstrated that collagens, especially fish collagens, have intriguing osteoconductive and biomechanical properties and are used more frequently in tissue engineering. Due to its exceptional biocompatibility, collagen has been reported to be employed as a biomaterial in a variety of vascular tissue applications. The bioactivity of collagen has caused this biopolymer to be widely used in skin tissue repair with its healing, antigenic, new-tissue-thickening, and adhesion properties.

One such technique is tissue engineering, which relies on the utilization of autologous chondrocytes and resorbable matrices. Visual acuity depends on a healthy cornea, which is the eye’s tough, transparent anterior surface. Damage to the cornea is a significant contributor to the lack of limbal stem cells that results in vision problems. To this goal, a number of treatment modalities are being created to address limbal stem cell insufficiency. The goal of this strategy was to create a biocompatible scaffold for growing limbal stem cells that completely replicate the human amniotic membrane. This was done by using a unique method based on fish collagen. It was discovered that the mechanical and physical forces of fish-scale-derived collagen were adequate for this purpose [153,154]. Collagen was also demonstrated to play a critical role in tooth tissue repair. Indeed, various collagen types retrieved using various procedures have demonstrated their ability to stimulate the regeneration of dental tissue; as a result, they can be employed in biomedical applications to regenerate tooth tissue [155,156].

Because of postoperative problems, including retears at the treated site, large and enormous rotator cuff tears pose a difficulty for surgeons. Since fish byproducts are regarded as a safer collagen source than other animal-derived scaffolds, collagen generated from fish scales has recently attracted more attention. Yamaura et al. [157] assessed the biological effectiveness of Tilapia-scales–derived collagen scaffolds for rotator cuff healing in rat models. In this research, by augmenting the repair site with a Tilapia-scale–derived collagen scaffold, after 6 weeks, an enhanced angiogenesis and fibrocartilage regeneration at the enthesis was observed. Due to osteogenic capacity and the connections between cells and the matrix, extracellular matrix and bioceramics are vital components in bone tissue regeneration. Since scaffolds are typically made up of synthetic polymers and bioceramics, surface modifications with hydrophilic materials, such as proteins, have great prospects for tissue engineering applications. In this study, which was provided by Kim et al. [158], marine atelocollagen was extracted from the bones and skins of *Paralichthys olivaceus*. Then, in vitro and in vivo calvarial implantation of the scaffolds with and without marine atelocollagen was performed to study bone tissue regeneration. The results of mineralization confirmed that scaffolds with marine atelocollagen showed an osteogenic increase from 300% to 1000% in different compositions, compared with pure scaffolds.

### 5.5. Wound Healing

**Figure 10:**
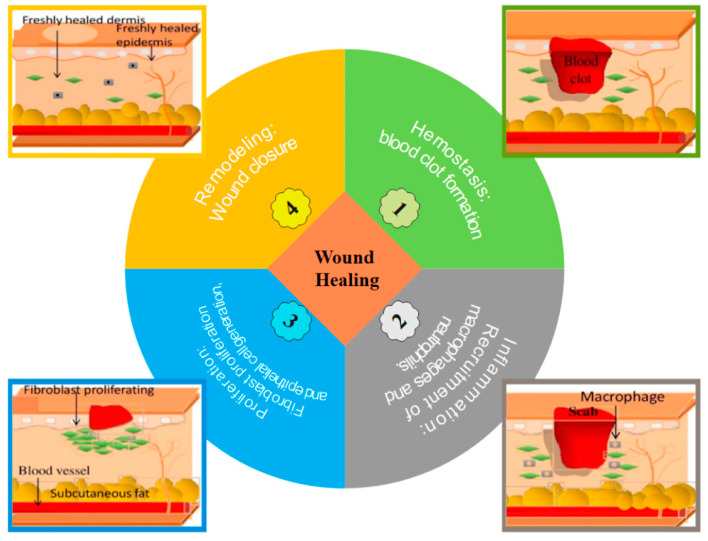
Schematic of the wound healing steps: (**1**) hemostasis, (**2**) inflammation, (**3**) proliferation, and (**4**) remodeling.

The complex process of wound healing is essential for re-establishing the skin’s barrier function. Numerous illnesses can halt this process, leaving behind chronic wounds that are extremely expensive to treat. Due to the complicated symptoms brought on by metabolic dysfunction of the wound microenvironment, such wounds fail to heal according to the stages of healing, and the comprehensive treatment of chronic wounds is still recognized as a huge unmet medical need. Consequently, there are three broad categories for wound classification: (i) superficial (involves only the epidermis), (ii) partial-thickness (involves epidermis and dermis), (iii) and full-thickness wound (involves also the underlying subcutaneous fat or deeper tissues) [159,160,161,162,163]. The process of wound healing is a physiological process that consists of four main steps: (i) hemostasis, (ii) inflammation, (iii) proliferation, and (iv) remodeling (Figure 10). Therefore, it is vital to choose the right polymers, bioactive chemicals, and wound dressings that can speed up the healing process. There is no one wound dressing that can be used to treat all types of wounds due to their varying etiology. Thus, the development of a smart wound dressing with antibacterial, anti-inflammatory, and antioxidant capabilities that, most critically, can benefit nearly all types of wounds, is the future challenge [163,164,165,166].

The combination of polymers and bioactive compounds significantly speeds up wound healing. Although the use of natural remedies for wound healing has been extensively studied, only a small number have yet to be commercialized or employed in clinical settings. In order to fully understand the potential of naturally occurring bioactive compounds in skin tissue regeneration, more preclinical studies must be done. Collagen, as a biodegradable organic tissue matrix, is a common option when choosing safe and nontoxic materials because it is one of the most crucial elements in tissue regeneration and wound healing and gives the skin its tensile strength. Collagen also has antimicrobial qualities and can aid in the hemostasis process. Collagen is used in different forms of hydrogel, sponge, and film for wound treatment. The best example of wound dressing devices are hydrogels, three-dimensional networks which can maintain a moist environment at the wound site and promote quicker tissue regeneration [161,164,167].

Several attempts at wound healing using prototypal devices made of fish-derived type I collagen or decellularized fish skin have been made. Hu et al. demonstrated that marine collagen peptides promote wound closure at concentrations of 50 μg.mL^−1^ commencing at 12 h after treatment with collagen using an in vitro scratch assay [168]. It was demonstrated that the cell migration that was induced was comparable to migration seen when using 10.0 μg/mL of epidermal growth factor, a factor known to be extremely important in wound healing. In addition, after 11 days, rabbits treated with marine collagen peptides extracted from the skin of Tilapia healed considerably quicker than the control group. Additionally, Yang et al. extracted collagen peptides from Alaska Pollock and showed that giving injured rats collagen peptides orally boosted recovery rates substantially more than those in the control groups [169]. Similarly, Chen et al. extracted collagen from bovine skin collagen nanofibers and marine Tilapia skin and demonstrated that collagen-treated rat groups recovered from wounds more quickly than control groups [170]. The study also discovered that collagen’s hydroxyproline, which promotes re-epithelization, has a significant influence in the rate of wound healing. In comparison to the control groups, the collagen-treated groups had more fibroblasts, higher vascularization, less inflammation, and more collagen fibers.

### 5.6. Food Packaging

Food packaging has the primary function of preserving and protecting food, primarily from oxidative and microbial degeneration, extending the shelf-life of the food by enhanced barrier and mechanical properties [171,172]. Fish collagen has attracted growing interest due to its potential for adding active and intelligent functions to conventional packaging [173,174]. In particular, active packaging can prevent the migration of H_2_O, O_2_, CO_2_, smells, and fats, and can include bioactive compounds such as antioxidants, antimicrobials, and taste to prolong the shelf life of the product [57,175,176]. Active packaging can appear in the form of edible films or coatings. Edible films are first produced by solution casting or compression molding and then applied to food surfaces by coating, wrapping, or spraying, while edible coatings are applied to food by spraying or dipping [177,178].

Films and coatings for food packaging must feature an elevated oxygen barrier and adequate thickness, mechanical properties, and transparency besides microbial stability, non-toxicity, and safety [179,180,181,182].

There are some necessary properties of biopolymers for food packaging, such as biodegradability, low water vapor permeability, oxygen barrier, thickness, transparency, edibility, and elasticity [183,184,185,186].

The application of fish collagen films is still constrained in the packaging industry due to drawbacks including poor mechanical qualities, low thermal stability, excessive water solubility and a large water vapor permeability. Several studies are in progress to overcome these limitations. For example, to reduce the brittleness, collagen films are usually prepared by using a plasticizer, mainly glycerol in the range 20–30 wt%, a small molecule of low volatility added to decrease attractive intermolecular forces along polymer chains and increase the free volume and chain mobility [187]. Moreover, suitable crosslinking treatments are being studied to improve the thermal stability of fish collagen [188,189]. Other possible solutions could be the blend of collagen with other biopolymers, mainly chitosan [77,190,191,192,193], and the addition of active compounds providing functional properties suitable for active packaging [187,194].

Gelatin, extracted from fish collagen by partial hydrolysis followed by thermal treatment, is attracting increasing interest for the development of edible films and coatings with probiotic properties, as recently reported in the literature [195,196,197]. In order to achieve the properties required for food packaging, several studies report on the physical or chemical modification of fish gelatin with chitosan, starch, soy protein isolate and carboxymethyl cellulose [198,199,200,201,202].

## 6. Collagen Market

Collagen and derivates are widely used for various applications, including dietary supplements, anti-aging formulations, soft-tissue growth devices, wound dressings, and food packaging. The achievement of US Food and Drug Administration (FDA) GRAS status (Generally Recognized as Safe) in 1983 for collagen and in 1975 for gelatin [203] boosted collagen’s use in several areas of application [204].

The increasing popularity of fish collagen for biomedical, food, cosmetic, nutraceutical, and nutricosmetic application has increased its demand. To this, the global marine collagen market was worth USD 685 million in 2020 [204] and USD 633 million in 2022 [205] and it was estimated to register over 5.3–7.5% of the compound annual growth rate (CAGR) between 2021 and 2029 [203,204] and is expected to reach a market size of USD 1123 million by 2032 [205]. In particular, the fish-collagen market was estimated to be worth USD 320.21 million in 2021 and is predicted to skyrocket to USD 624.12 million by 2029, with a CAGR of 8.7% during the forecast period 2022 to 2029 [206].

The increasing popularity of fish-collagen-based products is principally due to two main factors: (i) aging population, and (ii) environmental issues. The increase of the mean population age is directly correlated with the increase of age-related diseases (i.e., joint disorders, wrinkles, and wounds) [206,207]. In these circumstances, collagen-based products have been revealed to be effective, quite low-cost, easily accessible, safe, non-invasive, and readily available, and, accordingly, fish-derived-collagen awareness has significantly increased thanks to its additional advantages compared to other collagen types [208]. Therefore, the fish-collagen market for nutraceutical application was valued at over USD 280 million in 2020. Moreover, the rising inclination of consumers towards fat-free and nutritious products has further increased the product demand [204]. Thus, the major factor that is expected to boost the growth of the marine collagen market in the forecast period is a rise in the demand for supplements to control healthcare costs [203]. On the other hand, the environmental problems linked to the disposal of the enormous quantity of by-products of the fishing industry and to the use of plastic have shifted focus toward the search for eco-friendly solutions. In particular, waste recovery technologies were developed to reduce the environmental impact on by-products and to develop new products with added value. Local enterprises profited from this arrangement because fish is more readily available for less money, and the collagen market is booming [207]. Therefore, fish collagen and derivates started to be isolated, studied, and commercialized not only in health-related sectors but also in food packaging.

Fish collagen demand is related to its applications. In North America, it is mainly required for pharmaceutical applications [204]. In Europe and Australia, it its mainly used in the cosmetics industry [204,207]. The boost of fish collagen for cosmetic applications is principally due to the increasing preference for minimally or non-invasive surgical procedures compared to traditional surgical treatments. Additionally, the ease of treatment, the higher safety, and major accessibility have led the European population to prefer topical collagen formulations and food supplements for anti-aging and well-being treatments [209]. In Asia and Latin America, besides age-related issues, the major exploitation of fish collagen as a food supplement has arisen from the fact that, according to the European Nutraceutical Association (ENA), a lack of adequate nutrition accounted for 38.6% of deaths in China, India, and Brazil [204,207,209]. Indeed, the ENA’s in-depth investigation highlighted that inadequate nutrition is not related to an economic gap, but to incorrect eating habits [209].

**Figure 11:**
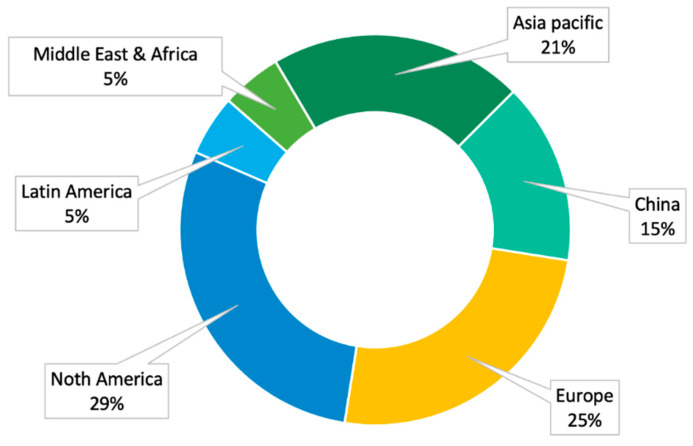
Fish-collagen market segmentation by Continent in 2019 [207].

Regarding countries’ contributions to the fish-collagen market, in 2012, the CARG of fish-collagen market by region was positive and was projected to reach +18% in North America, +31% in Latin America, +10% in Europe, and +28% in Asia by 2016 [209]. As shown in Figure 11, in 2019 North America (about 29%), Europe (about 30%), and Asia (Asia pacific: 21%, China: 15%) occupy the largest share of the market [209]. Among them, Asia is clearly expected to rule the market with about 36% of the total [207,209]. Actual fish-collagen market distribution by midlands is not available but it is known that North America’s contribution remained almost unchanged (31%) and that, in Europe, Germany contributes 23.3% to the total fish-collagen market, while, in Asia, Japan contributes 6.6% and, in Oceania, Australia’s contribution is about 2.6% [207].

The cost of fish-collagen is also application-related. The cost for the food industry (as binders, stabilizers, emulsifiers, film-formers, and fat replacers) was reported to be between EUR 8–12/kg, for the nutraceutical industry (for joint diseases) it was about EUR 10–12/kg, and for cosmetic applications it was reported to be about EUR 20–25/kg but could reach also EUR 40/kg [210]. However, the quality of the product obtained from marine life forms (USD 44539/metric ton) costs relatively higher than that from bovine sources (USD 33457/metric ton) [210] due to the complex and cost-intensive process of extracting collagen from marine organisms and by-products of the fishing industry. Moreover, fish waste has been somewhat decreased as a result of changes made to fishing regulations to combat overfishing, which limited the production of fish collagen and related goods. The high cost of fish collagen and the lack of awareness about its benefits among consumers are some of the major challenges faced by manufacturers [203]. These disadvantages allowed bovine collagen to have a leadership position as it holds a great cost advantage in lower-value products (e.g., food) [210].

The major players operating in the marine collagen market are Ajinomoto (Tokyo, Japan), Amicogen Deyan Biotech (Jinseong-myeon, South Korea), Ashland (Wilmington, CA, USA), Athos collagen (Surat, India), BDF Biotech (Girona, Spain), BHN (Tokyo, Japan), Certified Nutraceuticals (Pauma Valley, CA, USA), Cobiosa (Madrid, Spain), ETChem (Suzhou, China), Gelita (Eberbach, Germany), Juncà Gelatines (Girona, Spain), Hangzhou Nutrition Biotechnology (Hangzhou, China), HealthyHey Nutrition (Mumbai, India), HiMedia Laboratories (Maharashtra, India), Italgel (Cuneo, Italy), Lapi Gelatin (Empoly, Italy), Nippi Incorporated (Burnaby, Canada), Nitta Gelatin (Kokin, India), Norland Products (Jamesburg, NJ, USA), ProPlenish (Armadale, Australia), Rousselot (Gent, Belgium), Seagarden (Husøyvegen, Norway), Tessenderlo Group (Ixelles, Belgium), Weishardt Group (Graulhet, France), among others.

## 7. Challenges in the Industrial Implementation of Collagen Derived from Fish Waste

The collagen extraction process is a multistep, time-consuming procedure, which is a disadvantage in the industrial production of it. The issues and related challenges of fish collagen extraction are manifold and are principally linked to the extraction process and to the protein chemical-physical properties.

One of the main troubles is its low extraction yield, a parameter that is both species-related (i.e., taxonomy, age, tissue, and living conditions) and process-related (i.e., time, volumes, instrumentation, sample-volume ratio, types of acid and enzyme used and their concentrations, temperature, pH, ionic strength, and so on [211]). Several attempts were made in order to improve collagen extraction yield. The major steps forward have been made by optimizing solute and solvent concentrations and times in extraction steps 3–5. In particular, the implementation of a discarding phase of non-collagenous components (i.e., step 3 in Table 1) with NaOH 0.05–0.1 M, and an extraction phase (i.e., step 4 in Table 1) with an acetic acid concentration of 0.6 M for 36 h [212] brings a collagen yield increase. Regarding the enzymatic extraction, a pepsin concentration of 1200–1300 U/g is revered as the most effective in increasing collagen yield [98]. However, if, on one hand, the enzymatic extraction is able to significantly increase the yield of collagen, on the other hand, it significantly increases the time of the process and decreases the native conformation degree [213,214]. This consequence may not be industrially advantageous since it can lead to a higher cost of the process and therefore to a higher final cost of the product. For this reason, it is necessary to make a cost/benefit assessment before choosing whether or not to perform the enzymatic extraction. In addition to the ‘standard extraction process’ steps improvements, some innovative attempts have been made. Several authors demonstrated how the application of ultrasound increased yield and reduced processing time, as well as being greener compared to conventional extraction methods [79,109,213]. Huan et al. developed a novel rapid extrusion-hydro-extraction process for collagen from fish scales at room temperature [215] as an alternative to traditional methods.

Regardless of the process, temperature affects all extraction steps, from the tissue separation to the final collagen precipitation and recovery. Because of fish collagen’s low denaturation temperature (<37 °C), the need to carry out the entire extraction process at low temperatures (4–10 °C), to preserve its native structure and thus its structural properties and bioactivity, makes the procedure expensive. The low denaturation temperature of fish collagen is due to fish’s evolutionary adaptation to the characteristics of the aquatic environment in which they live. For this reason, it is not possible to intervene in this aspect. The only thing that can be done is to carefully select the fish species. In particular, the selection of a fish species that lives in a tropical environment—and therefore will have collagen with a physiologically higher denaturation temperature (e.g., 32–36 °C in catfish [215], 36–38 °C in carp [2,216], 32–37 °C in Tilapia [121], and 43 °C in lizardfish [217])—compared to a fish species living in cold waters, could be a solution. With this in mind, Pinedo et al. investigated the properties of collagen extracted from a hybrid fish line that, although similar to those of the original strains, was allowed to obtain a more controlled fish growth and, thus, a higher yield [81].

Despite the presence of various issues, it is clear how scientific and industrial research is moving towards the optimization of the extraction process and industrial implementation. In this regard, an advanced pilot plant automation was recently designed to maximize collagen extraction [218]. Therefore, since it is not possible to reduce the time and costs of the extraction process by optimizing it from the point of view of temperature control, a way to increase the denaturation temperature of marine collagen and make it more suitable for a wide range of applications is to induce post-synthesis crosslinking of the products. The increment of fish collagen denaturation temperature is another important issue since it is particularly relevant in some clinical applications. The application of crosslinking treatments also helps in the resolution of other two issues related to fish-collagen use which are the low mechanical properties and the low resistance to degradation, which make it unusable in some applications. Indeed, physical (e.g, UV [219,220], dehydrothermal treatment [219,221], chemical (e.g, methacrylation [222], pullulan [223], carbodiimide [221,224], N-hydroxysuccinimide-activated adipic acid [225]), and enzymatical (e.g., transglutaminase [225]) treatments were performed to enhance collagen properties. Maher et al. made a considerable step forward by successfully printing methacrylated fish collagen and realizing a 3D construct with desired properties, despite the fact that the applicated treatment was not able to increase the resistance to degradation on par with collagen extracted from mammals [222]. Another strategy commonly adopted to improve collagen properties is to blend it with other biomaterials with higher mechanical properties, such as chitosan [77,226,227,228], poly(lactic acid) [228,229], alginate [230], polyvinyl alcohol [227,231], and cellulose [195,231].

## 8. Conclusions

Natural biopolymers have unique biophysical and biochemical properties, including biocompatibility, biodegradability, increased body fluid adsorption capacity, increased gel-forming ability, non-toxic and non-immunogenic capabilities, as well as antifungal, antibacterial, and anticancer activities. One of these biopolymers is collagen, which could be obtained from various sources such as fish, mammalian, and agro-food waste. By turning these wastes into new products with a high functional value, recycling these by-products can assist in decreasing the pollution caused by these sorts of wastes. A potential substitute for bovine collagen is thought to be fish collagen. Fish collagen is cited as an important biomaterial due to its wide range of biological characteristics, including remarkable biocompatibility, high levels of cell adhesion, exceptional biodegradability, and low antigenicity. This review provides a general overview of collagen and its properties, types of sources and extraction methods, and diverse applications in a variety of industries, with a spotlight on fisheries and aquaculture sources.

## Figures and Tables

**Table 1:** Five steps of collagen extraction process from fish sources [88,89,90].

No.	Step(s)	Time
1	Separation and purification	2 h
2	Size reduction	1 h
3	Non-collagenous components discarding	Up to 3 days
4	Collagen extraction through acid and/or enzyme treatment	Up to 6 days
5	Salt precipitation and recovery	2 days

**Table 2:** Recent studies on various techniques for fish collagen extraction.

Source	Tissue	Extraction Method	Yield (%)	Reference
*Scomber japonicus*	Bone	Subcritical-water	1.75	[116]
	Skin		8.10	
*Thunnus obesus*	Skin	Acid (acetic acid)	13.5	[117]
	Skin	Enzymatic (pepsin)	16.7	
	Scale		4.6	
	Bone		2.6	
*Nibea japonica*	Swim bladders	Acid (acetic acid)	11.33	[118]
		Enzymatic + Acid (pepsin + acetic acid)	15.35	
*Cyprinus carpio*	Scale	Acid (hydrochloric acid, phosphoric acid, and sulfuric acid)	13.6	[114]
*Sardinella fimbriata*	Fringescale	Acid (acetic acid)	7.48	[119]
		Enzymatic (pepsin)	0.94	
*Hybrid Sturgeon*	Skin	Acid (hydrochloric acid, acetic acid, citric acid, and lactic acid)	5.73	[120]
		Enzymatic (pepsin)	10.26	
*Oreochromis niloticus*	Skin	Acid (acetic acid)	19.07	[121]
		Enzymatic + Acid (pepsin + acetic acid)	19.61	
*Acipenser schrenckii*	Skin	Enzymatic + Acid (pepsin + hydrochloric acid)	13.4	[122]
	Swim bladders		16.5	
	Notochord		1.7	
*Misgurnus* *anguillicaudatus*	Skin	Acid (acetic acid)	22.4	[123]
		Enzymatic (pepsin)	27.32	
*Miichthys miiuy*	Swim bladders	Acid	1.33	[124]
		Enzymatic (pepsin)	8.37	
*Saurida tumbil Bloch*	Skin	Acid (acetic acid)	11.73	[125]
		Acid (lactic acid)	11.63	
		Acid (citric acid)	11.39	
*Piaractus brachypomus*	Skin	Acid (acetic acid)	45.8	[126]
		Enzymatic (pepsin)	57.8	
*Nile Tilapia*	Skin	Acid (acetic acid)	4.76	[61]
		Enzymatic (pepsin)	8.14	
*Catla catla*	Skin	Acid (acetic acid)	63.40	[1,127]
		Enzymatic + Acid (pepsin + acetic acid)	69.53	
*Labeo rohita*	Skin	Acid (acetic acid)	46.13	
		Enzymatic + Acid (pepsin + acetic acid)	64.94	
*Thunnus obesus*	Skin	Enzymatic (bromelain)	3.05	[128]
		Enzymatic (papain)	42.76	
		Enzymatic (pepsin)	52.02	
		Enzymatic (trypsin)	13.83	

## Data Availability

Data available on request.
